# Concurrent Chemoradiotherapy with Daily Low-Dose Carboplatin in Older Patients with Unresectable Locally Advanced Non-Small-Cell Lung Cancer: Clinical Outcomes and Prognostic Significance of Systemic Inflammation Markers

**DOI:** 10.3390/curroncol33030135

**Published:** 2026-02-25

**Authors:** Yu Miura, Hisao Imai, Satoshi Endo, Kosuke Hashimoto, Ou Yamaguchi, Atsuto Mouri, Ken Masubuchi, Takeshi Masubuchi, Yuka Fujita, Shingo Kato, Hiroshi Kagamu, Kyoichi Kaira

**Affiliations:** 1 Department of Respiratory Medicine, International Medical Center, Saitama Medical University, 1397-1 Yamane, Hidaka 350-1298, Saitama, Japan; you_mi@saitama-med.ac.jp (Y.M.); hkosuke@saitama-med.ac.jp (K.H.); ouyamagu@saitama-med.ac.jp (O.Y.); mouria@saitama-med.ac.jp (A.M.); kagamu19@saitama-med.ac.jp (H.K.); kkaira@saitama-med.ac.jp (K.K.); 2 Division of Respiratory Medicine, Gunma Prefectural Cancer Center, 617-1 Takahayashinishi, Ota 373-8550, Gunma, Japan; endo-sa@gunma-cc.jp (S.E.); kmasubuchi@gunma-cc.jp (K.M.); tmasubuchi@gunma-cc.jp (T.M.); 3 Department of Respiratory Medicine, National Hospital Organization Asahikawa Medical Center, 7-4048 Hanasaki-cho, Asahikawa 070-8644, Hokkaido, Japan; fujita.yuka.gh@mail.hosp.go.jp; 4 Department of Radiation Oncology, International Medical Center, Saitama Medical University, 1397-1 Yamane, Hidaka 350-1298, Saitama, Japan; s_kato@saitama-med.ac.jp

**Keywords:** chemoradiotherapy, efficacy, Glasgow Prognostic Score, low-dose carboplatin, non-small-cell lung cancer

## Abstract

Older patients with unresectable locally advanced non-small-cell lung cancer (NSCLC) frequently receive concurrent chemoradiotherapy (CCRT) with daily low-dose carboplatin; however, real-world data on its efficacy, safety, and prognostic factors remain limited. We aimed to retrospectively evaluate the clinical outcomes of this regimen and examined whether systemic inflammation-based indices predict prognosis in this setting. We reviewed 52 consecutive patients with locally advanced NSCLC treated with first-line CCRT using daily low-dose carboplatin at three Japanese institutions between April 2007 and December 2019. The median progression-free survival (PFS) and overall survival (OS) were 11.5 and 40.1 months, respectively. Twenty patients received durvalumab as consolidation therapy. In the overall cohort, multivariate analysis identified the Glasgow Prognostic Score (GPS) as an independent predictor of PFS. A GPS of 0–1 was also associated with a significantly longer OS in univariate analysis. CCRT with daily low-dose carboplatin provided durable disease control with acceptable toxicity in older patients with unresectable stage II/III NSCLC. The GPS appears to be a simple marker for PFS in this population and may aid in pretreatment risk stratification alongside histology and consolidation strategies.

## Simple Summary

In Japan, older patients with unresectable locally advanced non-small-cell lung cancer (NSCLC) are commonly treated with concurrent chemoradiotherapy using daily low-dose carboplatin; however, evidence from real-world practice remains limited. In this multicenter retrospective study, we evaluated long-term clinical outcomes, safety, and prognostic significance of systemic inflammation markers in patients treated with this regimen. Among 52 older patients, chemoradiotherapy achieved durable disease control with acceptable toxicity. Notably, the Glasgow Prognostic Score (GPS), calculated from routinely measured C-reactive protein and albumin levels, emerged as a simple and objective predictor of progression-free survival, outperforming other inflammation-based indices. These findings suggest that daily low-dose carboplatin chemoradiotherapy is a feasible and effective option for older patients and that the GPS may serve as a practical tool for pretreatment risk stratification and individualized treatment planning in routine clinical practice.

## 1. Introduction

Lung cancer is the leading cause of cancer-related mortality worldwide [1]. Among these, unresectable, locally advanced non-small-cell lung cancer (NSCLC) represents approximately 30–35% of the overall NSCLC cases [2]. The disease predominantly affects older adults; the median age at diagnosis is 71 years, and nearly 70% of patients are aged >65 years at presentation [3,4]. Platinum-based concurrent chemoradiotherapy (CCRT) is the standard of care for individuals with unresectable locally advanced NSCLC. Nevertheless, its therapeutic impact is modest, with a reported median overall survival (OS) of only 22–25 months and a 5-year OS rate of approximately 20% [5].

The randomized phase 3 Japan Clinical Oncology Group (JCOG) 0301 trial, conducted by the JCOG, demonstrated that CCRT consisting of 60 Gy thoracic irradiation with daily low-dose carboplatin (30 mg/m^2^, 5 days per week for 4 weeks) provided superior survival and acceptable toxicity compared with radiotherapy alone in patients aged > 70 years with unresectable stage IIIA–IIIB NSCLC and performance status (PS) 0–2 [6]. Based on these findings, the current Japanese guidelines recommend daily low-dose carboplatin-based CCRT for older patients with unresectable stage II/III NSCLC. In addition, the pivotal phase 3 PACIFIC trial established that consolidation therapy with durvalumab following definitive chemoradiotherapy significantly improved progression-free survival (PFS) and OS compared with placebo [7,8]. Consequently, the National Comprehensive Cancer Network guidelines recommend durvalumab consolidation for patients with unresectable stage II/III NSCLC who maintain an Eastern Cooperative Oncology Group Performance Status (ECOG PS) of 0–1 and exhibit no disease progression after CCRT.

A substantial proportion of patients with lung cancer develop a systemic inflammatory response (SIR) and cancer-associated weight loss, both of which are central components of cancer cachexia [9,10]. Therefore, numerous SIR-based indices—including the Glasgow Prognostic Score (GPS), neutrophil-to-lymphocyte ratio (NLR), platelet-to-lymphocyte ratio (PLR), and Advanced Lung Cancer Inflammation Index (ALI)—have been evaluated as prognostic biomarkers. The GPS incorporates serum C-reactive protein (CRP) and albumin levels [9], and multiple studies have demonstrated its independent prognostic significance in advanced NSCLC [11,12,13,14,15,16]. However, the association between the GPS and treatment response to daily low-dose carboplatin-based CCRT in older patients with unresectable stage II/III NSCLC remains unclear. Similarly, although NLR has shown prognostic utility across various malignancies [17] and several studies have confirmed its prognostic relevance in NSCLC [18,19], its association with outcomes following CCRT in this specific population remains unclear. Systematic reviews have indicated that NLR predicts therapeutic efficacy and survival in NSCLC [20]. An elevated PLR, another marker of systemic inflammation, is associated with poor OS in lung cancer, particularly in NSCLC [21,22]. ALI, which reflects inflammatory and nutritional status, is also correlated with unfavorable OS in multiple cancer types [23], and baseline ALI is an independent predictor of poor prognosis in advanced NSCLC [24]. Moreover, prior work on body composition suggests that body mass index (BMI) influences survival outcomes in NSCLC [25,26]. Nevertheless, the potential prognostic associations between BMI, GPS, NLR, PLR, and ALI and therapeutic efficacy in older patients receiving daily low-dose carboplatin-based CCRT for unresectable stage II/III NSCLC have not yet been evaluated.

Therefore, this study aimed to investigate whether BMI, GPS, NLR, PLR, and ALI can serve as predictive markers for treatment effectiveness in patients receiving this regimen. Although daily low-dose carboplatin is widely used in Japan in older patients with unresectable stage II/III NSCLC, studies focusing on its real-world performance are scarce. Furthermore, the clinical effectiveness and toxicity profiles of this CCRT regimen in older patients have not been thoroughly characterized in real-world practice. Therefore, this retrospective analysis aimed to assess the safety and therapeutic efficacy of daily low-dose carboplatin-based CCRT in routine clinical care for older patients with unresectable stage II/III NSCLC and to help refine the treatment strategies for this population.

## 2. Materials and Methods

### 2.1. Participants

This retrospective study involved a comprehensive review of electronic medical records. We identified consecutive patients with unresectable stage II/III NSCLC who received CCRT with low-dose carboplatin between April 2007 and December 2019 at three Japanese institutions: Saitama Medical University International Medical Center, Gunma Prefectural Cancer Center, and National Hospital Organization Asahikawa Medical Center. Patients were required to have a minimum follow-up period of 60 months, with data censored on 31 December 2024. Follow-up was defined as the interval from the initiation of chemoradiotherapy to death or the date of last clinical contact. All patients had a minimum potential follow-up of 60 months by 31 December 2024; however, the actual observed follow-up varied according to the timing of events. The study protocol was approved by the Institutional Ethics Committee of Saitama Medical University International Medical Center (approval number 2024-048, 16 April 2025) and waived the need for written informed consent owing to the retrospective nature of this study. All the procedures conformed to the principles of the Declaration of Helsinki. This approval covered the retrospective collection and analysis of anonymized data from the collaborating institutions, and data-sharing agreements were established among all the participating centers.

Eligible patients met the following criteria: (i) cytological or histological confirmation of NSCLC, (ii) unilateral hemithoracic disease with regional nodal involvement suitable for treatment within a single radiation field, and (iii) receipt of first-line CRT. Patients diagnosed with stage II disease that was considered unresectable owing to medical comorbidities or technical factors were treated with definitive chemoradiotherapy in routine clinical practice. Histological subtyping followed the 2015 World Health Organization classification, and clinical staging was based on the Eighth Edition of the Union for International Cancer Control tumor-node-metastasis (TNM) system [2]. Adverse event severity was graded using the Common Terminology Criteria for Adverse Events version 5.0. Institutional standards for CRT eligibility typically included neutrophil count ≥ 1.5 × 10^3^/mm^3^, platelet count ≥ 1.0 × 10^5^/mm^3^, serum creatinine ≤ 1.5 mg/dL, total bilirubin ≤ 2.0 mg/dL, and transaminases ≤ 100 U/L.

Pretreatment evaluation for TNM staging consisted of a physical examination, chest radiography, contrast-enhanced computed tomography (CT) of the chest and abdomen, CT or magnetic resonance imaging of the brain, and bone scintigraphy or 18F-fluorodeoxyglucose positron emission tomography.

### 2.2. Treatment

#### 2.2.1. Chemotherapy

Chemotherapy selection, in accordance with approved product labeling, was determined by the treating physicians. The patients received daily intravenous low-dose carboplatin (30 mg/m^2^ infused over 30 min) administered 1 h before radiotherapy for the initial 20 fractions. Treatment was discontinued in cases of disease progression, unacceptable toxicity, or withdrawal of consent. After durvalumab received regulatory approval, eligible patients received durvalumab as consolidation therapy 1–42 days following CRT completion. In patients with unresectable stage II NSCLC, durvalumab was administered at the discretion of the treating physician, reflecting real-world clinical practice despite limited prospective evidence. Durvalumab was intravenously administered at a dose of 10 mg/kg every 2 weeks for up to 12 months.

#### 2.2.2. Radiotherapy

Radiotherapy was administered according to the JCOG0301 protocol [6]. Briefly, radiotherapy was administered to the thoracic lesion once daily, 5 days per week, over 6–9 weeks. The total dose to the planning target volume was 60 Gy delivered in 30 fractions. Three-dimensional conformal radiotherapy and intensity-modulated radiotherapy were accepted to use with the institutional practice. Respiratory motion management strategies were implemented as necessary. Appropriate image guidance using on-board images, portal vision, and/or cone-beam CT was performed during the treatment session.

Regarding dose constraints for organs at risk, lung V20 < 30%, heart V40 < 20%, and maximum spinal cord doses < 45 Gy represented institutional planning goals. These constraints were not applied as strict eligibility criteria, and patients were not excluded from treatment or analysis based solely on deviations from these constraints.

### 2.3. Assessment of Metabolic and Inflammatory Indices

The baseline BMI was calculated as body weight (kg) divided by height squared (m^2^). Following established Japanese population data, a BMI cutoff of 22.0 kg/m^2^ was applied (high BMI ≥ 22.0, low BMI < 22.0) [27]. Serum CRP and albumin levels measured on the day before or on the day of CRT initiation were used to compute the GPS, which was categorized as follows:

0: CRP < 1.0 mg/dL and albumin ≥ 3.5 mg/dL

1: CRP elevation or hypoalbuminemia

2: CRP ≥ 1.0 mg/dL and albumin < 3.5 mg/dL [9,10]

The NLR was defined as the absolute neutrophil count divided by the absolute lymphocyte count, with a cutoff of 5.0 to differentiate low-risk (<5.0) and high-risk (≥5.0) groups [28,29]. The PLR was calculated similarly, with a cutoff of 185 used to classify patients into low-risk (<185) and high-risk (≥185) categories [21]. The ALI was defined as (BMI × albumin)/NLR, with a cutoff of 24 distinguishing low (<24) from high (≥24) ALI groups [23].

### 2.4. Evaluation of the Treatment Response

Tumor response was assessed based on the best overall response and maximum tumor shrinkage. Responses were classified according to the Response Evaluation Criteria in Solid Tumors, version 1.1 [30], as complete response, partial response, stable disease, progressive disease, or not evaluable. In case of treatment failure, subsequent therapy was selected at the discretion of the treating physician.

### 2.5. Statistical Analyses

Categorical variables were analyzed using Fisher’s exact test, and continuous variables were analyzed using Welch’s *t*-test. PFS was defined as the interval from treatment initiation to disease progression or death from any cause, whereas OS was defined as the interval from treatment initiation to death or last follow-up. Survival curves were estimated using the Kaplan–Meier method and compared using the log-rank test. Variables with *p*-value < 0.10 in univariate analysis were entered into the multivariate Cox proportional hazards model. Hazard ratios and 95% confidence intervals were calculated. Missing data were handled by performing complete-case analysis. The proportional hazards assumption was assessed and found to be acceptable. Statistical significance was set at a two-sided *p*-value < 0.05. All analyses were performed using the JMP software, version 11.0 (SAS Institute, Cary, NC, USA).

## 3. Results

### 3.1. Patient Characteristics and Treatment Efficacy

Fifty-two patients were included in this study. Patient characteristics are summarized in Table 1(A), while laboratory and inflammation-related indices are shown in Table 1(B).

The cohort comprised 41 men (78.8%) and 11 women (21.2%), with a median age of 76 (range, 71–86) years. Most patients (51/52, 98.1%) had an ECOG PS of 0–1, whereas one (1.9%) patient had a PS of 2. Adenocarcinoma and squamous cell carcinoma were observed at equal frequencies (23 patients each, 44.2%). Seven (13.5%) patients harbored driver alterations (*EGFR*, *ALK*, or *ROS1*), whereas the remaining patients were either negative or untested, and no additional oncogenic alterations were identified. All patients with stage II disease had unresectable tumors and were not candidates for curative surgery. The median BMI was 22.2 (range, 16.1–27.6) kg/m^2^. Two (3.8%) patients were unable to complete the radiotherapy, and the median delivered dose was 60 Gy (range, 45–66 Gy). Twelve (23.1%) patients discontinued carboplatin prematurely, and the median number of carboplatin administrations was 20 (range, 4–20). At the data cutoff, 16 (30.8%) patients were alive. Twenty (38.5%) patients received durvalumab consolidation therapy, and 32 (61.5%) did not. Among the 20 patients receiving durvalumab, 13 (65.0%) were unable to complete 1 year of treatment. Causes of discontinuation included disease progression (6/13, 46.2%) and treatment-related adverse events (7/13, 53.8%) (Table S1). Patterns of distant recurrence following CRT are listed in Table S2. Among the 40 patients who developed distant metastases, the bone (12 patients, 30.0%) and brain (6 patients, 15.0%) were the most frequent sites. Table S3 summarizes the systemic therapies administered after recurrence. Treatment response data are presented in Table 2.

The overall response rate (ORR) was 51.9% (95% confidence interval [CI], 38.6–64.8%). There were no statistically significant differences in either the ORR or disease control rate between patients who received durvalumab consolidation therapy and those who did not.

### 3.2. Survival Analysis

**Figure 1:**
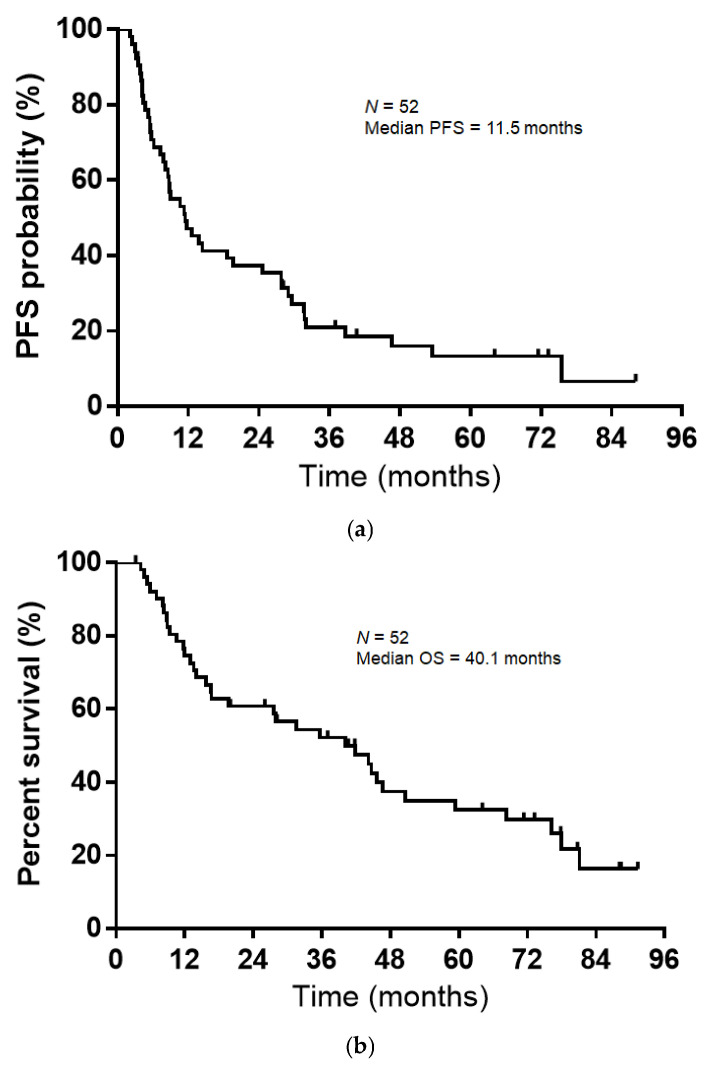
(**a**) Kaplan–Meier curves for progression-free survival (PFS) among 52 patients who received chemoradiotherapy. The median PFS period was 11.5 months (95% confidence interval [CI], 8.1–24.5). (**b**) Kaplan–Meier curves for overall survival (OS) among 52 patients who received chemoradiotherapy. The median OS period was 40.1 months (95% CI, 16.6–50.6).

The median follow-up duration was 29.8 months (range, 3.4–91.3 months), and the median PFS and OS were 11.5 months (95% CI, 8.1–24.5) and 40.1 months (95% CI, 16.6–50.6), respectively (Figure 1a,b). Patients who received durvalumab consolidation therapy showed numerically longer PFS compared with those who did not; however, the difference was not statistically significant. No significant difference in OS was observed between the two groups.

At the data cutoff (31 December 2024), 36 patients died and 16 survived. Univariate and multivariate analyses of PFS and OS are summarized in Table 3.

**Figure 2:**
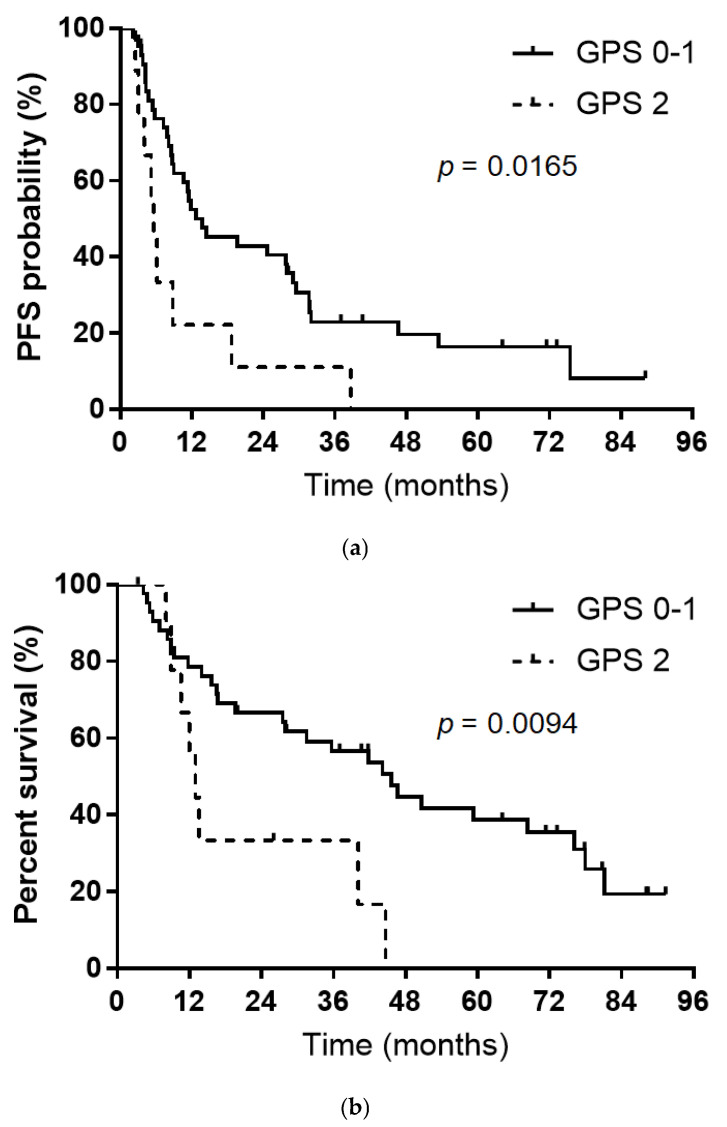
(**a**) Progression-free survival (PFS) according to Glasgow Prognostic Score (GPS) at the initiation of chemoradiotherapy (GPS 0–1, median PFS = 13.7 months; GPS 2, median PFS = 5.6 months). (**b**) Overall survival (OS) according to Glasgow Prognostic Score (GPS) at the initiation of chemoradiotherapy (GPS 0–1, median OS = 45.6 months; GPS 2, median OS = 13.0 months).

In the univariate analysis, GPS was significantly associated with PFS. Multivariate analysis confirmed that GPS (0–1 vs. 2) was an independent factor for PFS (hazard ratio, 0.36; *p* = 0.0294). Univariate analysis identified significant associations between histology and GPS; however, multivariate analysis did not identify any independent predictors of OS. The Kaplan–Meier survival curves are shown in Figure 2.

Patients with a GPS of 0–1 had significantly longer PFS and OS than those with a GPS of 2 (both *p* < 0.05). The median PFS periods were 13.7 months for GPS 0–1 and 5.6 months for GPS 2 (log-rank *p* = 0.0165, Figure 2a), and the median OS periods were 45.6 and 13.0 months, respectively (log-rank *p* = 0.0094, Figure 2b).

### 3.3. Toxicity

Treatment-related adverse events are shown in Table 4.

All 52 patients were evaluable for safety. Myelosuppression was the most common toxicity, with grade 3–4 decreased white blood cell counts in 25.0% of the patients, grade 3–4 decreased neutrophil counts in 23.1%, and febrile neutropenia in one (1.9%). Grade 3–4 decreased platelet counts were observed in nine (17.3%) patients. Severe nonhematological toxicities were rare; grade 3–4 skin rashes occurred in 3.8% of the patients. Treatment discontinuation due to adverse events occurred in 12 of the 52 (23.1%) patients. No treatment-related deaths occurred. Among the 20 patients receiving durvalumab consolidation therapy, 7 discontinued treatment because of adverse events, including 6 cases of pneumonitis and 1 of myositis (Table S1).

### 3.4. Subsequent Treatment After Chemoradiotherapy

Post-CRT treatments are summarized in Table S3. Of the 40 patients who experienced recurrence, best supportive care was frequently selected; however, 26 (65.0%) received systemic therapy. Nivolumab monotherapy was the most common subsequent regimen, followed by platinum-based combination chemotherapy. Nine patients received up to third-line therapy, and four patients received more than four lines of treatment.

## 4. Discussion

This study assessed the real-world efficacy and safety of CCRT with daily low-dose carboplatin in older patients with unresectable stage II/III NSCLC and evaluated the prognostic utility of BMI, GPS, NLR, PLR, and ALI. Multivariate analysis identified the GPS as being an independent predictor of PFS, highlighting its potential as a clinically meaningful biomarker. To the best of our knowledge, this is the first study to explore the prognostic relevance of these inflammation- and nutrition-based indices, specifically in patients receiving daily low-dose carboplatin CCRT for unresectable locally advanced NSCLC. Notably, this study included patients with unresectable stage II/III disease and those with postoperative recurrence. Although these populations differ in terms of disease biology and prior treatment history, all patients received definitive concurrent chemoradiotherapy using the same daily low-dose carboplatin regimen in routine clinical practice. Therefore, they were analyzed together to identify real-world treatment patterns.

Findings from the JCOG0301 randomized phase 3 trial demonstrated that adding daily low-dose carboplatin to thoracic radiotherapy provided significant clinical benefits with acceptable toxicity in older patients with unresectable stage II/III NSCLC, yielding a median PFS period of 8.9 months and a median OS period of 22.4 months [6]. In our cohort, the median PFS and OS periods were 11.5 months and 40.1 months, respectively—outcomes that appear more favorable than those reported in JCOG0301. Several factors may explain this discrepancy. Our cohort included patients who received durvalumab consolidation therapy (n = 20), individuals with stage II disease (n = 4), patients experiencing postoperative recurrence (n = 7), and patients who subsequently received modern systemic therapies not available during the JCOG0301 trial period—including third-generation EGFR tyrosine kinase inhibitors (TKIs), second-generation ALK TKIs, and immune checkpoint inhibitor monotherapy. These agents have been demonstrated to have substantial efficacy in advanced NSCLC [31,32,33,34,35,36] and likely contributed to the improved OS observed in this study. The effect of durvalumab maintenance may also have influenced PFS prolongation. Furthermore, the relatively favorable OS observed in this cohort should be interpreted in the context of the long study enrollment period. During the study period, substantial advances occurred in radiotherapy techniques, supportive care, and systemic therapies after disease progression, including molecular targeted agents and immune checkpoint inhibitors.

The PACIFIC trial showed that durvalumab maintenance following platinum-based CCRT significantly extended PFS and OS compared with placebo, with median PFS periods of 16.8 and 5.6 months and median OS periods of 47.5 and 29.1 months, respectively [7,8]. In our analysis, patients receiving durvalumab consolidation therapy exhibited a numerical, but not statistically significant, improvement in PFS and OS. There are several possible explanations for these results. First, our cohort was considerably older (median age, 76 years) than the PACIFIC population (median age, 64 years), consistent with the PACIFIC subgroup analysis in which age > 65 years was correlated with a diminished OS benefit [37]. Another potential factor is the chemoradiation regimen. The PACIFIC trial did not administer a daily, low-dose carboplatin regimen. Although a retrospective study provided real-world data on durvalumab consolidation maintenance after chemoradiotherapy [38], including patients who received a daily low-dose carboplatin regimen, no focused analysis was performed on this population. Therefore, the clinical significance of durvalumab consolidation therapy following chemoradiotherapy with low-dose carboplatin remains unclear.

However, a Japanese phase II study (NEJ039A) reported that durvalumab consolidation therapy after chemoradiotherapy with daily low-dose carboplatin was effective and well-tolerated in older patients with unresectable locally advanced NSCLC [39]. In this study, the median PFS and OS periods were 12.3 and 28.1 months, respectively, and the average follow-up period from the first enrollment was 19.0 months. Although the median observation periods differed and direct comparison with that study’s results was not possible, the PFS was similar to that in our cohort.

A recent retrospective study comparing patients who received durvalumab consolidation therapy after CCRT with daily low-dose carboplatin versus those who did not suggested that durvalumab consolidation therapy after CCRT with daily low-dose carboplatin did not provide a significant clinical benefit in older patients with unresectable stage II/III NSCLC [40]. This was a small, retrospective study that compared 16 patients who received durvalumab consolidation therapy with 20 patients who did not. Owing to this previous study’s small sample size, it is possible that no statistically significant difference was observed. Our current analysis, which compared 20 patients who received durvalumab consolidation therapy with 32 who did not, was also a small-sample retrospective study and may have yielded similar results. Because durvalumab became available during the latter part of the study period, patient selection for consolidation therapy may have been influenced by treatment era, PS, and post-chemoradiotherapy toxicities. Therefore, comparisons between patients treated with and without durvalumab should be interpreted with caution.

The role of durvalumab consolidation therapy after chemoradiotherapy was established in the PACIFIC trial [7,8]; however, evidence in older patients treated with daily low-dose carboplatin remains limited. In the present real-world cohort, patients who received durvalumab showed numerically longer PFS compared with those who did not, although the difference was not statistically significant. This finding should be interpreted cautiously given the small sample size resulting in limited statistical power and retrospective study design. Beyond the PACIFIC trial, several real-world studies have examined the feasibility and outcomes of durvalumab consolidation therapy following concurrent chemoradiotherapy. The PACIFIC-R study and other retrospective cohort analyses have demonstrated that durvalumab consolidation is generally feasible in routine practice [38,41,42]. However, real-world completion rates are often lower than those reported in clinical trials, particularly among older patients. Treatment discontinuation owing to immune-related adverse events, especially pneumonitis, is more frequent in older or frailer populations than in the PACIFIC cohort [42]. In our study, the completion rate of durvalumab consolidation therapy was 35%, and discontinuation was primarily driven by progressive disease and pneumonitis. These findings are consistent with real-world studies reporting that tolerability and treatment adherence in older patients may differ from that in trial populations.

The present study was not intended to definitively evaluate the efficacy of durvalumab consolidation therapy, and no causal conclusions should be drawn. Notably, our study population was substantially older than that of the PACIFIC trial, and a relatively high proportion of patients discontinued durvalumab due to treatment-related adverse events, particularly pneumonitis. These results suggest that while durvalumab consolidation is feasible in selected older patients, careful patient selection, close monitoring, and early management of immune-related toxicities are essential in routine clinical practice. In the present study, durvalumab use in patients with unresectable stage II disease reflected real-world clinical decision-making rather than strict guideline-based recommendations, and its efficacy in this subgroup should be interpreted with caution. Ongoing and recent Japanese prospective trials, such as JCOG1914, are investigating optimized chemoradiotherapy and immunotherapy strategies in older patients with locally advanced NSCLC [43]. Compared with these trials, our study provides complementary real-world data with long-term follow-up, thereby reflecting routine clinical practice rather than protocol-driven treatment.

Among the inflammation- and nutrition-based indices evaluated in this study, the GPS has emerged as an independent prognostic factor of PFS. Studies have demonstrated the prognostic significance of the GPS in NSCLC, irrespective of the disease stage, and it is commonly used as a prognostic marker in clinical research [10,11,12,13,14,15,16]. The GPS is associated with altered drug metabolism, adipokine levels, elevated cytokine levels, weight loss, muscle wasting, and compromised PS [10,44,45,46,47,48,49]. Previous studies have evaluated the GPS in the context of first-line cytotoxic drug treatment for NSCLC, whereas no study has evaluated the GPS with respect to CCRT with daily low-dose carboplatin. In the present study, the GPS 2 group (n = 9) was smaller than the GPS 0–1 group (n = 43). This may be because locally advanced NSCLC, unlike NSCLC with distant metastases, is characterized by lesions confined to the unilateral thoracic region.

Although PS has long been considered a primary prognostic factor in clinical trials and real-world practice, its assessment is inherently subjective and dependent on the physician’s judgment. In the population analyzed here, only one of the 52 patients had a PS of 2, precluding the evaluation of patients with poor PS. Moreover, most patients in this study had good performance status (ECOG PS 0–1), reflecting clinical selection in routine practice. Therefore, the present findings are most applicable to relatively fit older patients and should not be directly extrapolated to frailer populations. In contrast to the PS, which is subjective and dependent on physician assessment, the GPS is an objective and highly reproducible index of baseline patient condition, stratifying patients using a simple three-point scoring system. This objectivity may allow for a more accurate and standardized pretreatment risk assessment than conventional prognostication based on PS alone [50]. The GPS is calculated from serum CRP and albumin levels, both of which can be routinely and easily measured in daily clinical practice in most medical institutions; thus, the GPS reflects both systemic inflammation and nutritional status and can be easily assessed in routine clinical practice. Multivariate analysis demonstrated that the GPS was independently associated with PFS (Table 3). Importantly, the GPS should be regarded as a prognostic marker reflecting host-related factors rather than a predictive marker for sensitivity to a specific chemoradiotherapy regimen. Notably, a GPS of 2, defined by elevated CRP levels and hypoalbuminemia at treatment initiation, was associated with inferior treatment efficacy, suggesting that systemic inflammation and nutritional status substantially influence the outcomes. These findings support the clinical integration of the GPS into treatment decision-making for patients with unresectable stage II/III NSCLC receiving CCRT with daily low-dose carboplatin.

In contrast, other inflammation-related and nutrition-related indices, including NLR, PLR, ALI, and BMI, were not independently associated with survival in the multivariate analyses. Although prior studies on advanced NSCLC have suggested the prognostic relevance of these markers, the results have been inconsistent, likely reflecting heterogeneous patient populations, variable treatment settings, and differing cutoff values [18,19,21,22,24,25,26]. In particular, patients with locally advanced NSCLC without distant metastases may exhibit less pronounced systemic inflammation than those with metastatic NSCLC, potentially reducing the discriminatory power of these indices in this clinical context. Furthermore, BMI has been reported as a prognostic factor in advanced NSCLC, particularly in patients treated with immune checkpoint inhibitors, in whom a higher BMI is associated with improved survival [25,26]. However, in the present cohort of patients with unresectable locally advanced disease treated with definitive chemoradiotherapy, BMI was not significantly associated with either PFS or OS. This discrepancy may reflect differences in disease stage, treatment modality, and patient population. In locally advanced NSCLC without distant metastases, baseline BMI alone may have limited prognostic impact compared with systemic inflammatory and nutritional markers. The lack of independent prognostic significance for NLR, PLR, ALI, and BMI may be attributable to the limited sample size that may have limited the statistical power, variability in optimal cutoff values, and clinical characteristics of locally advanced disease without distant metastases, which may be associated with less pronounced systemic inflammation. Future investigations in larger, well-defined cohorts treated with uniform CCRT regimens are required to establish optimal cutoff values and account for ethnicity-related differences, particularly with respect to BMI, when extrapolating these indices beyond Japanese populations.

Daily low-dose carboplatin treatment was well-tolerated. No grade ≥ 3 pneumonitis was observed, in contrast with the 1% incidence reported in JCOG0301. Rates of grade ≥ 3 hematological toxicities and febrile neutropenia were also lower than those observed in JCOG0301. No treatment-related death occurred during the study period. These findings suggest that daily low-dose carboplatin combined with thoracic radiotherapy is a safe and feasible treatment regimen for older patients. Among 20 patients receiving durvalumab consolidation therapy, seven discontinued treatment owing to adverse events, predominantly pneumonitis (n = 6). Although no grade 4–5 events were observed, the high rate of pneumonitis-related discontinuation warrants caution when administering durvalumab consolidation therapy to older patients after low-dose carboplatin CCRT.

This study has some limitations. First, this study had a limited sample size. The small sample size limited the statistical power and may have obscured differences between clinical subgroups. Additionally, the introduction of modern systemic therapies, including durvalumab consolidation, during the study period may have influenced long-term outcomes in ways that cannot be fully disentangled. The long study period spanning pre- and post-immunotherapy eras may have confounded survival outcomes, especially OS, and this effect could not be fully adjusted for owing to the limited sample size. Furthermore, owing to the limited sample size, subgroup or sensitivity analyses excluding postoperative recurrence were not feasible. The limited representation of patients with poor PS further restricts the generalizability of the results. Second, the retrospective design introduced potential variability in clinical assessments and outcome documentation. Additionally, because this was a retrospective multicenter study, radiotherapy planning and dose distributions may have differed between institutions. Third, patient selection may have been influenced by institutional treatment policies, which may have introduced selection bias. The inclusion of heterogeneous clinical populations, including postoperative recurrence cases, may also have influenced survival outcomes and the interpretation of prognostic factors.

## 5. Conclusions

CCRT with low-dose carboplatin is an effective and well-tolerated treatment option for older patients with unresectable stage II/III NSCLC. The GPS is a simple, objective, and clinically valuable predictor of PFS, outperforming other inflammation-based indices and offering a practical tool for pretreatment risk stratification. Although durvalumab consolidation therapy is feasible, a survival benefit is not clearly observed in this older population. Collectively, these findings support the continued use of daily low-dose carboplatin CCRT in routine practice and highlight the importance of incorporating the GPS into individualized treatment planning for older patients with unresectable stage II/III NSCLC.

## Figures and Tables

**Table 1:** (**A**) Patient characteristics. (**B**) Laboratory and inflammation-related indices.

(A)							
**Characteristic**	Total (n = 52)	(%)	With Durvalumab (n = 20)	(%)	Without Durvalumab (n = 32)	(%)	*p*-Value
*Sex*							
Men	41	78.8	17	85.0	24	75.0	0.49
Women	11	21.2	3	15.0	8	25.0	
*Age (years)*							
Median	76		76		76		0.23 **
Range	71–86		71–80		71–86		
*Performance status (ECOG-PS)*							
0	29	55.8	9	45.0	20	62.5	0.28 ***
1	22	42.3	11	55.0	11	34.4	
2	1	1.9	0	0	1	3.1	
*Smoking status*							
Current or former	43	82.7	17	85.0	26	81.3	>0.99
Never	9	17.3	3	15.0	6	18.7	
*Histology*							
Adenocarcinoma	23	44.2	8	40.0	15	46.9	0.31 ***
Squamous cell carcinoma	23	44.2	8	40.0	15	46.9	
Others	6	11.6	4	20.0	2	6.2	
*Driver mutations/translocations (EGFR, ALK, ROS-1)*							
Positive	7	13.5	1	5.0	6	18.7	-
Wild type or negative	37	71.1	15	75.0	22	68.8	
Others	0	0	0	0	0	0	
Not tested	8	15.4	4	20.0	4	12.5	
*PD-L1 TPS (%)*							
<1	11	21.2	6	30.0	5	15.6	-
1–49	13	25.0	7	35.0	6	18.7	
≥50	9	17.3	3	15.0	6	18.7	
Unknown	19	36.5	4	20.0	15	46.9	
*Disease stage*							
II	4	7.7	3	15.0	1	3.1	0.27 ***
III	41	78.8	15	75.0	26	81.3	
Postoperative recurrence	7	13.5	2	10.0	5	15.6	
*History of postoperative adjuvant chemotherapy*							
Yes	0	0	0	0	0	0	>0.99
No	52	100	20	100	32	100	
*BMI (kg/m^2^)*							
Median	22.2		20.6		23.3		0.017 **
Range	16.1–27.6		16.1–26.8		18.2–27.6		
*Radiotherapy planned dose completion*							
Yes	50	96.2	20	100	30	93.8	0.51
No	2	3.8	0	0	2	6.2	
*Irradiation dose (Gy)*							
Median	60		60		60		0.51 **
Range	45–66		60		45–66		
*Administration of CBDCA planned dose completion*							
Yes	40	76.9	14	70.0	26	81.3	0.5
No	12	23.1	6	30.0	6	18.7	
*Number of cycles CBDCA administered*							
Median	20		20		20		0.37 **
Range	4–20		6–20		4–20		
*Reason for discontinuation of CBDCA administration*							
Progressive disease	0	0	0	0	0	0	-
Adverse events	11	21.2	6	30.0	5	15.6	
Worsening of PS	0	0	0	0	0	0	
Others	1	1.9	0	0	1	3.1	
(**B**)							
**Characteristic**	**Total (n = 52)**	**(%)**	**With Durvalumab** **(n = 20)**	**(%)**	**Without Durvalumab** **(n = 32)**	**(%)**	***p*** **-Value**
*Laboratory data, median* [*range*]							
CRP (mg/dL)	0.3 (0.0–10.1)		0.2 (0.0–10.1)		0.3 (0.0–6.3)		0.56 **
Albumin (g/dL)	3.7 (2.0–4.5)		3.8 (2.0–4.4)		3.7 (2.3–4.5)		0.93 **
Neutrophil (cells/mm^3^)	4274 (2103–8116)		4371 (2103–8116)		4274 (2188–7200)		0.39 **
Lymphocyte (cells/mm^3^)	1279 (530–10,150)		1203 (729–10,150)		1371 (530–2160)		0.38 **
Platelets (cells/mm^3^)	246,000 (116,000–514,000)		256,000 (131,000–514,000)		234,000 (116,000–336,000)		0.23 **
*GPS*							
0, 1	43	82.7	17	85.0	26	81.3	>0.99
2	9	17.3	3	15.0	6	18.7	
*NLR*							
Low (<5)	44	84.6	15	75.0	29	90.6	0.23
High (≥5)	8	15.4	5	25.0	3	9.4	
*PLR*							
Low (<185)	23	44.2	6	30.0	17	53.1	0.15
High (≥185)	29	55.8	14	70.0	15	46.9	
*ALI*							
Low (<24)	26	50.0	14	70.0	12	37.5	0.004
High (≥24)	26	50.0	6	30.0	20	62.5	
*Relapse at data cutoff*							
Yes	44	84.6	15	75.0	29	90.6	0.23
No	8	15.4	5	25.0	3	9.4	
*Alive at data cutoff*							
Alive	16	30.8	8	40.0	8	25.0	0.35
Death	36	69.2	12	60.0	24	75.0	

ECOG, Eastern Cooperative Oncology Group; PS, performance status; PD-L1, programmed death-ligand 1; TPS, tumor proportion score; BMI, body mass index; CBDCA, carboplatin; CRP, C-reactive protein; GPS, Glasgow Prognostic Score; NLR, neutrophil-to-lymphocyte ratio; PLR, platelet-to-lymphocyte ratio; ALI, Advanced Lung Cancer Inflammation Index. ** Welch’s *t*-test. *** Chi-squared test.

**Table 2:** Comparison of treatment responses between the overall population and patients with and without durvalumab consolidation therapy.

Response	Total (n = 52)	(%)	With Durvalumab (n = 20)	Without Durvalumab (n = 32)	*p*-Value
Complete response	0	0	0	0	
Partial response	27	51.9	10	17	
Stable disease	22	42.3	10	12	
Progressive disease	3	5.8	0	3	
Not evaluated	0	0	0	0	
Response rate, % (95% CI)		51.9 (38.6–64.8)	50.0 (28.0–71.9)	53.1 (35.8–70.4)	>0.99
Disease control rate, % (95% CI)		94.2 (83.7–98.6)	100	90.6 (80.5–100)	0.27

CI, confidence interval.

**Table 3:** Associations between clinical factors and progression-free survival (PFS) and overall survival (OS).

		Univariate Analysis			Multivariate Analysis				Univariate Analysis			Multivariate Analysis		
		PFS			PFS				OS			OS		
Factors	Median PFS (Months)	HR	95% CI	*p*-Value	HR	95% CI	*p*-Value	Median OS (Months)	HR	95% CI	*p*-Value	HR	95% CI	*p*-Value
Sex														
Men/women	10.6/24.5	1.1	0.55–2.37	0.78				35.7/44.1	0.91	0.44–2.07	0.82			
Age (years) at the start of chemoradiotherapy														
71–74/≥75	8.8/11.8	1.12	0.54–2.17	0.73				16.7/45.6	1.79	0.80–3.69	0.14			
Smoking status														
Current or former/never	11.3/13.7	1.05	0.51–2.46	0.88				27.9/50.6	1.42	0.63–3.78	0.41			
Histology														
Adenocarcinoma/non-adenocarcinoma	13.7/8.8	0.84	0.46–1.53	0.58				59.4/16.6	0.48	0.24–0.95	0.0353			
Driver mutations/translocations														
Positive/negative or unknown	11.3/11.8	1.37	0.55–2.93	0.46				51.8/31.5	0.89	0.33–2.01	0.80			
Disease stage at diagnosis														
II–III/postoperative recurrence	12.6/8.5	0.74	0.31–2.18	0.55				41.8/27.5	1.48	0.45–4.86	0.51			
BMI (kg/m^2^)														
Low (<22.0)/high (≥22.0)	10.4/11.5	1.04	0.57–1.89	0.88				40.1/41.8	1.24	0.64–2.41	0.50			
GPS														
0, 1/2	13.7/5.6	0.41	0.20–0.92	**0.0329**	0.36	0.16–0.89	**0.0294**	45.6/13.0	0.34	0.15–0.84	**0.0218**	0.42	0.16–1.18	0.09
NLR														
Low (<5)/high (≥5)	12.6/7.3	0.58	0.27–1.45	0.23	0.55	0.20–1.60	0.26	44.1/13.3	0.42	0.17–1.17	0.09	0.66	0.22–2.13	0.47
PLR														
Low (<185)/high (≥185)	11.3/12.6	1.05	0.56–1.93	0.85	0.98	0.47–2.08	0.96	45.6/31.5	0.83	0.42–1.61	0.59	1.13	0.50–2.55	0.76
ALI														
Low (<24)/high (≥24)	10.4/11.5	0.74	0.40–1.34	0.32	0.49	0.21–1.07	0.07	13.3/45.6	1.53	0.78–2.98	0.20	1.2	0.50–2.78	0.66
Durvalumab consolidation therapy														
Yes/no	24.3/10.6	0.61	0.31–1.12	0.11				40.1/41.8	1.02	0.48–2.04	0.95			

The bold font indicates a statistically significant difference. PFS, progression-free survival; OS, overall survival; HR, hazard ratio; CI, confidence interval; BMI, body mass index; GPS, Glasgow Prognostic Score; NLR, neutrophil-to-lymphocyte ratio; PLR, platelet-to-lymphocyte ratio; ALI, Advanced Lung Cancer Inflammation Index.

**Table 4:** Adverse events during chemoradiotherapy.

Adverse Event	Any Grade	%	Grade ≥ 3	%
Led to discontinuation	12	23.1	10	19.2
Led to death	-	-	0	0
Treatment-related adverse events				
White blood cell decreased	-	-	13	25.0
Neutrophil count decreased	-	-	12	23.1
Platelet count decreased	-	-	9	17.3
Febrile neutropenia	-	-	1	1.9
Skin rash	-	-	2	3.8
Liver dysfunction	-	-	1	1.9
Infection	-	-	1	1.9

Excluding the adverse events leading to discontinuation of treatment, only grade 3 or higher adverse events are described here.

## Data Availability

The data generated in this study are available upon request from the corresponding author. The data are not publicly available due to ethical restrictions.

## Supplementary Materials

The following supporting information can be downloaded at https://www.mdpi.com/article/10.3390/curroncol33030135/s1, Table S1. Durvalumab consolidation therapy; Table S2. Sites of metastases at recurrence; Table S3. Subsequent treatment of 40 patients with recurrence after chemoradiotherapy.

## Abbreviations

The following abbreviations are used in this manuscript:

ALI	Advanced Lung Cancer Inflammation Index
BMI	body mass index
CCRT	concurrent chemoradiotherapy
CI	confidence interval
CRP	C-reactive protein
CT	computed tomography
GPS	Glasgow Prognostic Score
JCOG	Japan Clinical Oncology Group
NLR	neutrophil-to-lymphocyte ratio
NSCLC	non-small-cell lung cancer
ORR	overall response rate
OS	overall survival
PLR	platelet-to-lymphocyte ratio
PS	performance status
SIR	systemic inflammatory response
TKIs	tyrosine kinase inhibitors
TNM	tumor-node-metastasis

## References

[1] Bray F., Laversanne M., Sung H., Ferlay J., Siegel R.L., Soerjomataram I., Jemal A. (2024). Global cancer statistics 2022: GLOBOCAN estimates of incidence and mortality worldwide for 36 cancers in 185 countries. CA Cancer J. Clin..

[2] Goldstraw P., Chansky K., Crowley J., Rami-Porta R., Asamura H., Eberhardt W.E.E., Nicholson A.G., Groome P., Mitchell A., Bolejack V. (2016). The IASLC lung cancer staging project: Proposals for revision of the TNM stage groupings in the forthcoming (eighth) edition of the TNM classification for lung cancer. J. Thorac. Oncol..

[3] Venuta F., Diso D., Onorati I., Anile M., Mantovani S., Rendina E.A. (2016). Lung cancer in elderly patients. J. Thorac. Dis..

[4] Presley C.J., Reynolds C.H., Langer C.J. (2017). Caring for the older population with advanced lung cancer. Am. Soc. Clin. Oncol. Educ. Book.

[5] Hanna N., Neubauer M., Yiannoutsos C., McGarry R., Arseneau J., Ansari R., Reynolds C., Govindan R., Melnyk A., Fisher W. (2008). Phase III study of cisplatin, etoposide, and concurrent chest radiation with or without consolidation docetaxel in patients with inoperable stage III non-small-cell lung cancer: The Hoosier Oncology Group and U.S. Oncology. J. Clin. Oncol..

[6] Atagi S., Kawahara M., Yokoyama A., Okamoto H., Yamamoto N., Ohe Y., Sawa T., Ishikura S., Shibata T., Fukuda H. (2012). Thoracic radiotherapy with or without daily low-dose carboplatin in elderly patients with non-small-cell lung cancer: A randomised, controlled, phase 3 trial by the Japan Clinical Oncology Group (JCOG0301). Lancet Oncol..

[7] Antonia S.J., Villegas A., Daniel D., Vicente D., Murakami S., Hui R., Yokoi T., Chiappori A., Lee K.H., de Wit M. (2017). Durvalumab after chemoradiotherapy in Stage III non-small-cell lung cancer. N. Engl. J. Med..

[8] Antonia S.J., Villegas A., Daniel D., Vicente D., Murakami S., Hui R., Kurata T., Chiappori A., Lee K.H., de Wit M. (2018). Overall survival with durvalumab after chemoradiotherapy in Stage III NSCLC. N. Engl. J. Med..

[9] McMillan D.C. (2008). An inflammation-based prognostic score and its role in the nutrition-based management of patients with cancer. Proc. Nutr. Soc..

[10] Proctor M.J., Talwar D., Balmar S.M., O’Reilly D.S., Foulis A.K., Horgan P.G., Morrison D.S., McMillan D.C. (2010). The relationship between the presence and site of cancer, an inflammation-based prognostic score and biochemical parameters. Initial results of the Glasgow Inflammation Outcome Study. Br. J. Cancer.

[11] Forrest L.M., McMillan D.C., McArdle C.S., Angerson W.J., Dunlop D.J. (2004). Comparison of an inflammation-based prognostic score (GPS) with performance status (ECOG) in patients receiving platinum-based chemotherapy for inoperable non-small-cell lung cancer. Br. J. Cancer.

[12] Gioulbasanis I., Pallis A., Vlachostergios P.J., Xyrafas A., Giannousi Z., Perdikouri I.E., Makridou M., Kakalou D., Georgoulias V. (2012). The Glasgow Prognostic Score (GPS) predicts toxicity and efficacy in platinum-based treated patients with metastatic lung cancer. Lung Cancer.

[13] Leung E.Y., Scott H.R., McMillan D.C. (2012). Clinical utility of the pretreatment Glasgow prognostic score in patients with advanced inoperable non-small cell lung cancer. J. Thorac. Oncol..

[14] Jiang A.G., Chen H.L., Lu H.Y. (2015). Comparison of Glasgow prognostic score and prognostic index in patients with advanced non-small cell lung cancer. J. Cancer Res. Clin. Oncol..

[15] Takamori S., Takada K., Shimokawa M., Matsubara T., Fujishita T., Ito K., Toyozawa R., Yamaguchi M., Okamoto T., Yoneshima Y. (2021). Clinical utility of pretreatment Glasgow prognostic score in non-small-cell lung cancer patients treated with immune checkpoint inhibitors. Lung Cancer.

[16] Imai H., Kishikawa T., Minemura H., Yamada Y., Ibe T., Yamaguchi O., Mouri A., Hamamoto Y., Kanazawa K., Kasai T. (2021). Pretreatment Glasgow prognostic score predicts survival among patients with high PD-L1 expression administered first-line pembrolizumab monotherapy for non-small cell lung cancer. Cancer Med..

[17] Templeton A.J., McNamara M.G., Šeruga B., Vera-Badillo F.E., Aneja P., Ocaña A., Leibowitz-Amit R., Sonpavde G., Knox J.J., Tran B. (2014). Prognostic role of neutrophil-to-lymphocyte ratio in solid tumors: A systematic review and meta-analysis. J. Natl. Cancer Inst..

[18] Liu Z.L., Zeng T.T., Zhou X.J., Ren Y.N., Zhang L., Zhang X.X., Ding Z.Y. (2016). Neutrophil–lymphocyte ratio as a prognostic marker for chemotherapy in advanced lung cancer. Int. J. Biol. Markers.

[19] Liu D., Jin J., Zhang L., Li L., Song J., Li W. (2018). The neutrophil to lymphocyte ratio may predict benefit from chemotherapy in lung cancer. Cell. Physiol. Biochem..

[20] Platini H., Ferdinand E., Kohar K., Prayogo S.A., Amirah S., Komariah M., Maulana S. (2022). Neutrophil-to-lymphocyte ratio and platelet-to-lymphocyte ratio as prognostic markers for advanced non-small-cell lung cancer treated with immunotherapy: A systematic review and meta-analysis. Medicina.

[21] Ding N., Pang Z., Shen H., Ni Y., Du J., Liu Q. (2016). The prognostic value of PLR in lung cancer, a meta-analysis based on results from a large consecutive cohort. Sci. Rep..

[22] Kinoshita A., Onoda H., Imai N., Iwaku A., Oishi M., Fushiya N., Koike K., Nishino H., Tajiri H. (2012). Comparison of the prognostic value of inflammation-based prognostic scores in patients with hepatocellular carcinoma. Br. J. Cancer.

[23] Hua X., Chen J., Wu Y., Sha J., Han S., Zhu X. (2019). Prognostic role of the advanced lung cancer inflammation index in cancer patients: A meta-analysis. World J. Surg. Oncol..

[24] Jafri S.H., Shi R., Mills G. (2013). Advance lung cancer inflammation index (ALI) at diagnosis is a prognostic marker in patients with metastatic non-small cell lung cancer (NSCLC): A retrospective review. BMC Cancer.

[25] Ichihara E., Harada D., Inoue K., Sato K., Hosokawa S., Kishino D., Watanabe K., Ochi N., Oda N., Hara N. (2020). The impact of body mass index on the efficacy of anti-PD-1/PD-L1 antibodies in patients with non-small cell lung cancer. Lung Cancer.

[26] Imai H., Naito E., Yamaguchi O., Hashimoto K., Iemura H., Miura Y., Shiono A., Mouri A., Kaira K., Kobayashi K. (2022). Pretreatment body mass index predicts survival among patients administered nivolumab monotherapy for pretreated non-small cell lung cancer. Thorac. Cancer.

[27] Tokunaga K., Matsuzawa Y., Kotani K., Keno Y., Kobatake T., Fujioka S., Tarui S. (1991). Ideal body weight estimated from the body mass index with the lowest morbidity. Int. J. Obes..

[28] Bagley S.J., Kothari S., Aggarwal C., Bauml J.M., Alley E.W., Evans T.L., Kosteva J.A., Ciunci C.A., Gabriel P.E., Thompson J.C. (2017). Pretreatment neutrophil-to-lymphocyte ratio as a marker of outcomes in nivolumab-treated patients with advanced non-small-cell lung cancer. Lung Cancer.

[29] Suh K.J., Kim S.H., Kim Y.J., Kim M., Keam B., Kim T.M., Kim D.W., Heo D.S., Lee J.S. (2018). Post-treatment neutrophil-to-lymphocyte ratio at week 6 is prognostic in patients with advanced non-small cell lung cancers treated with anti-PD-1 antibody. Cancer Immunol. Immunother..

[30] Eisenhauer E.A., Therasse P., Bogaerts J., Schwartz L.H., Sargent D., Ford R., Dancey J., Arbuck S., Gwyther S., Mooney M. (2009). New response evaluation criteria in solid tumours: Revised RECIST guideline (version 1.1). Eur. J. Cancer.

[31] Ramalingam S.S., Vansteenkiste J., Planchard D., Cho B.C., Gray J.E., Ohe Y., Zhou C., Reungwetwattana T., Cheng Y., Chewaskulyong B. (2020). Overall survival with osimertinib in untreated, EGFR-mutated advanced NSCLC. N. Engl. J. Med..

[32] Peters S., Camidge D.R., Shaw A.T., Gadgeel S., Ahn J.S., Kim D.W., Ou S.I., Pérol M., Dziadziuszko R., Rosell R. (2017). Alectinib versus crizotinib in Untreated ALK-Positive non-small-Cell Lung Cancer. N. Engl. J. Med..

[33] Shaw A.T., Bauer T.M., de Marinis F., Felip E., Goto Y., Liu G., Mazieres J., Kim D.W., Mok T., Polli A. (2020). First-line lorlatinib or crizotinib in advanced ALK-positive lung cancer. N. Engl. J. Med..

[34] Camidge D.R., Kim H.R., Ahn M.J., Yang J.C.H., Han J.Y., Lee J.S., Hochmair M.J., Li J.Y.C., Chang G.C., Lee K.H. (2018). Brigatinib versus crizotinib in ALK-Positive non-small-Cell Lung Cancer. N. Engl. J. Med..

[35] Borghaei H., Gettinger S., Vokes E.E., Chow L.Q.M., Burgio M.A., de Castro Carpeno J., Pluzanski A., Arrieta O., Frontera O.A., Chiari R. (2021). Five-year outcomes from the randomized, Phase III trials CheckMate 017 and 057: Nivolumab versus docetaxel in previously treated non-small-cell lung cancer. J. Clin. Oncol..

[36] Herbst R.S., Baas P., Kim D.W., Felip E., Pérez-Gracia J.L., Han J.Y., Molina J., Kim J.H., Arvis C.D., Ahn M.J. (2016). Pembrolizumab versus docetaxel for previously treated, PD-L1-positive, advanced non-small-cell lung cancer (KEYNOTE-010): A randomised controlled trial. Lancet.

[37] Spigel D.R., Faivre-Finn C., Gray J.E., Vicente D., Planchard D., Paz-Ares L., Vansteenkiste J.F., Garassino M.C., Hui R., Quantin X. (2022). Five-year survival outcomes from the PACIFIC trial: Durvalumab after chemoradiotherapy in Stage III non-small-cell lung cancer. J. Clin. Oncol..

[38] Girard N., Bar J., Garrido P., Garassino M.C., McDonald F., Mornex F., Filippi A.R., Smit H.J.M., Peters S., Field J.K. (2023). Treatment characteristics and real-world progression-free survival in patients with unresectable Stage III NSCLC who received durvalumab after chemoradiotherapy: Findings from the PACIFIC-R study. J. Thorac. Oncol..

[39] Mouri A., Kisohara A., Morita R., Ko R., Nakagawa T., Makiguchi T., Isobe K., Ishikawa N., Kondo T., Akiyama M. (2024). A phase II study of daily carboplatin plus irradiation followed by durvalumab therapy for older adults (≥75 years) with unresectable III non-small-cell lung cancer and performance status of 2: NEJ039A. ESMO Open.

[40] Yamauchi K., Komuta R., Tanabe H., Yokoyama M., Takata S.O., Yanase T., Hosono Y., Satoh S., Morishita N., Suzuki H. (2025). Real-world outcomes of durvalumab consolidation in elderly patients with unresectable NSCLC following CCRT with daily low-dose carboplatin. Anticancer Res..

[41] Huang Y., Zhao J.J., Soon Y.Y., Wong A., Aminkeng F., Ang Y., Asokumaran Y., Low J.L., Lee M., Choo J.R.E. (2022). Real-world experience of consolidation durvalumab after concurrent chemoradiotherapy in stage III non-small cell lung cancer. Thorac. Cancer.

[42] Park J.E., Hong K.S., Choi S.H., Lee S.Y., Shin K.C., Jang J.G., Kwon Y.S., Park S.H., Choi K.J., Jung C.Y. (2024). Durvalumab consolidation after chemoradiotherapy in elderly patients with unresectable Stage III NSCLC: A real-world multicenter study. Clin. Lung Cancer.

[43] Shimoyama R., Omori S., Nomura S., Kenmotsu H., Takahashi T., Harada H., Ishikura S., Mizutani T., Ando M., Kataoka T. (2021). A multi-institutional randomized phase III study comparing weekly carboplatin plus nab-paclitaxel and daily low-dose carboplatin as regimens for concurrent chemoradiotherapy in elderly patients with unresectable locally advanced non-small cell lung cancer: Japan Clinical Oncology Group Study JCOG1914. Jpn. J. Clin. Oncol..

[44] Brown D.J., Milroy R., Preston T., McMillan D.C. (2007). The relationship between an inflammation-based prognostic score (Glasgow Prognostic Score) and changes in serum biochemical variables in patients with advanced lung and gastrointestinal cancer. J. Clin. Pathol..

[45] Kerem M., Ferahkose Z., Yilmaz U.T., Pasaoglu H., Ofluoglu E., Bedirli A., Salman B., Sahin T.T., Akin M. (2008). Adipokines and ghrelin in gastric cancer cachexia. World J. Gastroenterol..

[46] Giannousi Z., Gioulbasanis I., Pallis A.G., Xyrafas A., Dalliani D., Kalbakis K., Papadopoulos V., Mavroudis D., Georgoulias V., Papandreou C.N. (2012). Nutritional status, acute phase response and depression in metastatic lung cancer patients: Correlations and association prognosis. Support. Care Cancer.

[47] Naito T., Tashiro M., Yamamoto K., Ohnishi K., Kagawa Y., Kawakami J. (2012). Impact of cachexia on pharmacokinetic disposition of and clinical responses to oxycodone in cancer patients. Eur. J. Clin. Pharmacol..

[48] McMillan D.C. (2013). The systemic inflammation-based Glasgow Prognostic Score: A decade of experience in patients with cancer. Cancer Treat. Rev..

[49] Kim S.J., Ryu K.J., Hong M., Ko Y.H., Kim W.S. (2015). The serum CXCL13 level is associated with the Glasgow Prognostic Score in extranodal NK/T-cell lymphoma patients. J. Hematol. Oncol..

[50] Dajczman E., Kasymjanova G., Kreisman H., Swinton N., Pepe C., Small D. (2008). Should patient-rated performance status affect treatment decisions in advanced lung cancer?. J. Thorac. Oncol..

