# Supplementary material for: Concurrent Chemoradiotherapy with Daily Low-Dose Carboplatin in Older Patients with Unresectable Locally Advanced Non-Small-Cell Lung Cancer: Clinical Outcomes and Prognostic Significance of Systemic Inflammation Markers
Source: Curr Oncol. 2026 Feb 25;33(3):135. doi: 10.3390/curroncol33030135 (PMC13026046; doi:10.3390/curroncol33030135)
Supplement: Supplementary file 1 [file curroncol-33-00135-s001.zip › curroncol-4113302-supplementary.pdf]

Supplementary material

Table S1. Durvalumab consolidation therapy.

**Characteristic**	**n=20**	**(%)**
Completion of 1 year of durvalumab consolidation therapy		
Yes	7	35.0
No	13	65.0
Reason for discontinuation of durvalumab consolidation therapy		
Progressive disease	6	46.2
Adverse events	7	53.8
Pneumonitis	6	
Myositis	1	

Table S2. Sites of metastases at recurrence.

Characteristic	n=40	(%)
Intracranial metastases at initial treatment		
Yes	6	15.0
No	34	85.0
Liver metastases at initial treatment		
Yes	2	5.0
No	38	95.0
Bone metastases at initial treatment		
Yes	12	30.0
No	28	70.0

Table S3. Subsequent treatment of 40 patients with recurrence after chemoradiotherapy.

Chemotherapeutic regimen	Second-line	Third-line	≥Fourth-line	Total
Platinum combination	4	2	0	6
Docetaxel	2	4	0	6
Pemetrexed	1	1	0	2
S1	0	1	0	1
Gefitinib	2	0	0	2
Osimertinib	3	0	0	3
Alectinib	1	0	0	1
Crizotinib	1	0	0	1
Lorlatinib	0	0	1	1
Brigatinib	0	0	1	1
Ipilimumab plus nivolumab	3	0	0	3
Nivolumab	8	0	3	11
Pembrolizumab	1	0	0	1
Atezolizumab	0	1	0	1
Investigational agent	0	0	0	0
Best supportive care	14	-	-	14
